# Correlation between clinical parameters characterising peri-implant and periodontal health: A practice-based research in Spain in a series of patients with implants installed 4-5 years ago

**DOI:** 10.4317/medoral.17999

**Published:** 2012-05-01

**Authors:** Roberto Lopez-Piriz, Araceli Morales, Maria J. Giménez, Antonio Bowen, Rafael Carroquino, Lorenzo Aguilar, Ignacio Corral, Cora del Val, Inmaculada González, Luis M. Ilzarbe, Juan R. Maestre, Esteban Padullés, Francisco Torres-Lear, Juan J. Granizo, Fide San-Román, Sofía Hernández, José Prieto

**Affiliations:** 1 Sociedad Española de Implantes, Madrid, Spain; 2 Microbiology Department, School of Medicine, Universidad Complutense, Madrid & PRISM-AG, Madrid, Spain; 3 School of Dentistry, Universidad Alfonso X El Sabio, Madrid, Spain; 4 School of Dentistry, Universidad de Zaragoza, Zaragoza, Spain; 5 Grana Datos, Madrid, Spain; 6 Surgery Department, School of Veterinary, Universidad Complutense, Madrid, Spain

## Abstract

Objectives: To explore periimplant health (and relation with periodontal status) 4-5 years after implant insertion. Study Design: A practice-based dental research network multicentre study was performed in 11 Spanish centres. The first patient/month with implant insertion in 2004 was considered. Per patient four teeth (one per quadrant) showing the highest bone loss in the 2004 panoramic X-ray were selected for periodontal status assessment. Bone losses in implants were calculated as the differences between 2004 and 2009 bone levels in radiographs. Results: A total of 117 patients were included. Of the 408 teeth considered, 73 (17.9%) were lost in 2009 (losing risk: >50% for bone losses ≥7mm). A total of 295 implants were reviewed. Eight of 117 (6.8%) patients had lost implants (13 of 295 implants installed; 4.4%). Implant loss rate (quadrant status) was 1.4% (edentulous), 3.6% (preserved teeth), and 11.1% (lost teeth) (p=0.037). The percentage of implant loss significantly (p<0.001) increased when the medial/distal bone loss was ≥3 mm. The highest (p≤0.001) pocket depths were found in teeth with ≥5mm and implants with ≥3mm bone losses, with similar mean values (≥4mm), associated with higher rates of plaque index and bleeding by probing. Conclusions: The significant bi-directional relation between plaque and bone loss, and between each of these two parameters/signs and pocket depths or bleeding (both in teeth and implants, and between them) together with the higher percentage of implants lost when the bone loss of the associated teeth was ≥3 mm suggest that the patient’s periodontal status is a critical issue in predicting implant health/lesion.

** Key words:**Implants, periimplantitis, periodontitis, oral health, practice-based research

## Introduction

Periodontitis can be considered the consequence of broken balances in bacterial components of the plaque (1). Its prevalence drives to its consideration as the most prevalent infectious disease in the community (2), with 75% of adults affected as reported in published studies (3,4). Several studies have identified similarities in the pathogenesis of late periodontitis and peri-implantitis, showing intra-oral translocation of periodontal pathogens from teeth showing chronic periodontitis to the peri-implant niche (5), producing at last the lost of affected teeth or implants. Previous history of periodontitis, poor oral hygiene and smoking are considered risk factors for peri-implantitis, and late dental implant failures are associated with peri-implantitis and/or biomechanical forces (6). While peri-implantitis is defined on implant basis (an inflammatory process leading to deformation of the peri-implant pocket and bone loss around an implant in function (7), periodontitis is defined on subject basis (individuals with more than one tooth (8) showing alterations not only in the classical measures of bone loss but also in additional parameters as bleeding on probing and probing pocket depth) (9).

Practice-based dental research networks have been used to identify problems in “real-life” dental practice (10). In restorative dentistry they have the potential to become as important for improvements in clinical practice as laboratory research is to knowledge of basic science (11).

The aim of this practice-based dental research network study was to explore peri-implant health (and its relation with the periodontal status) 4-5 years after implant insertion, using parameters measured in daily practice (bone loss, plaque index, pocket depth and bleeding on probing) in a series of patients not selected based on clinical diagnoses.

## Material and Methods

A multicentre study was carried out in 2009 in 11 Spanish dental clinics to assess implant health conditions in patients with at least one implant installed in 2004. To avoid selection bias, centres were asked to include the first patient of each month (except August) with implant insertion in 2004. Patients were contacted by phone and were asked to voluntarily participate by attending the clinic for a revision including X-ray. If the patient was not able or declined to attend the visit, the second patient of the month with implant insertion was contacted. The protocol from the Spanish Society for Implants (Sociedad Española de Implantes; SEI) was followed, and a case report form was used to collect data from 2004 (from clinical records) and 2009 (in the revision visit). Examiners (one per centre) were instructed for data collection to minimize inter-center variations. The study protocol was approved by the Ethics Committee of Hospital Clinico Universitario San Carlos, Madrid, Spain (CP-CI 10/140-E).

Demographic data, general health conditions, co-morbidities (diabetes, cardiovascular disease, immunosupression…), habits (smoking, alcohol intake), treatment with biphosphonates, and dental bruxism were noted from clinical records in 2004 and updated in 2009. The four teeth (one per quadrant and not removed for implant insertion) showing the highest bone loss in the panoramic radiograph performed in 2004 prior to implant insertion were used as index sites for the assessment of the periodontal status of each patient. Bone levels at their mesial and distal aspects were determined by assessing the distance between the most coronal position of the supporting bone and the cement-enamel junction with the use of a transparent ruler scaled (12). If the cement-enamel junction (CEJ) was masked due to crown restorations, the level was estimated by a connecting line between the CEJ and the neighbouring teeth. In the case of edentulous quadrants, data were recorded for the remaining quadrants.

Data from teeth and implants recorded at the revision visit in 2009 consisted in: plaque index (categorized as no plaque, plaque present on probing or visible plaque using the Plaque Index modified by Mombelli (mPI) (13), pocket probing depth (mesio-vestibular -MV-, vestibular -V-, distovestibular -DV-, mesio-lingual -ML-, lingual -L- and disto-lingual -DL-), mobility, bleeding on probing (using the modified sulcus bleeding index) (14), pain and suppuration. In addition, for implants, position, mesial and distal crestal bone levels, implant characteristics, and use or not of regenerative techniques were also recorded. Intraoral radiographs with a standardised paralleling technique (15) were used for implant bone level assessment. The distance between the implant platform (implant-abutment junction) and the bone implant contact at the mesial and distal aspects of each implant were measured in 2004 and 2009. The implant bone loss was calculated as the difference between vertical bone levels in the intraoral X-ray in 2004 (at abutment connection) and levels in the intraoral X-ray in 2009, both for mesial and distal measures. Frequency of consultation attendance was also recorded.

Case report forms were sent for double data entry using SPSS v.14 (SPSS Inc., Chicago, Il., USA). Comparisons of quantitative variables were performed by the t-test or ANOVA tests, using Tuckey test for comparisons of two groups. Correlations between quantitative variables were calculated by the Spearman (non-parametric) correlation test. Qualitative variables were compared by the Chi-square or Fisher’s exact test when necessary. Multivariate analyses were performed by multiple linear regression in order to explain the role of bone loss (both as quantitative variable and categorized as ≤1 mm; >1-<2 mm and ≥2 mm for implants and <3 mm; 3-<5 mm and ≥5 mm for teeth) and plaque index (categorized as no plaque, plaque present on probing and visible plaque) on pocket probing depths as dependent variable. An independent analysis was performed for each of the six measuring sites of pocket probing depths, and both for teeth and implants. In order to avoid false associations in multiple comparisons, p<0.01 was considered significant.

## Results

Characteristics of patients

Nine centres included 11 patients each, one centre included 10 patients and the remaining centre included eight patients. A total of 147 patients were phoned and 30 patients failed to be contacted or refused to participate. A total of 117 patients (mean age 56.3 ± 11.8 years; 37.6% males) were included in the study. Of them, 37.6% were smokers, 17.1% ex-smokers, 26.5% presented mild to moderate alcohol intake, 24.9% bruxism, 6.0% diabetes and 4.3% cardiovascular disease. A total of 64 (54.7%) patients had attended the previous annual dental revisions.

In 2004, 84.6% patients presented teeth in the four quadrants, 1.7% patients in three quadrants, 4.3% patients in two quadrants, 0.9% patients in one quadrant, and 8.5% patients were completely edentulous.

The risk of losing teeth

A total of 408 teeth were identified in the panoramic X-ray performed in 2004 as those with the highest bone loss per quadrant: 30.1% were at position 7, 21.6% at 6, 13.0% at 5, and 35.3% at other positions. There was a significant correlation between values of bone loss at mesial and distal sites, both globally and per quadrant (r2≥0.73; p≤0.001). Correlations were also found between mesial values (r2≥0.63; p≤0.001) in the four quadrants, as well as between distal values (r2≥0.55; p≤0.001). Globally, values of bone loss at distal sites were higher than those at mesial sites (3.77 ± 2.04 vs. 3.61 ± 2.07), although differences tended to be significant only in the upper quadrants (p=0.003 for quadrant 1 and p=0.05 for quadrant 2). Of the 408 teeth identified for the study (present in 2004), 73 (17.9%) had been lost when patients attended the revision in 2009. No relations could be found between tooth loss and the recorded demographic or health conditions, although the relation between tooth loss and bruxism was almost significant (p=0.012) since higher number of patients among those that lost teeth from 2004 to 2009 presented bruxism (40.5% vs. 17.7%).

**Figure 1:**
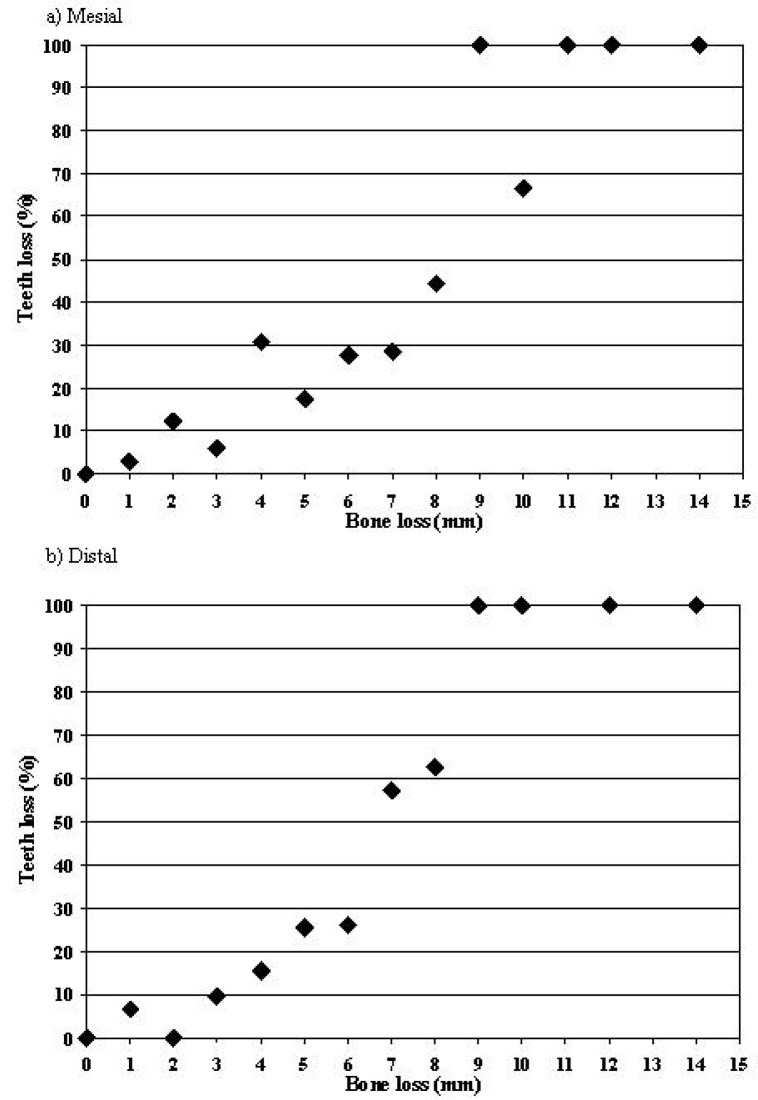
Risk of losing teeth in relation to bone loss in 2004.

Fig. 1 shows the percentage of tooth loss in relation to distal and mesial bone loss in 2004. The adjusted odds ratio of losing teeth linked to each mm was 1.77 (95%CI=1.50-2.08) for the distal bone loss and 1.56 (95%CI=1.36-1.79) for the mesial bone loss. As shown in the figure, the risk of losing teeth was >50% when distal bone losses were ≥7 mm. Correlations between the number of teeth lost and bone loss were significant (r2≥0.47; p≤0.001) for upper quadrants and distal bone loss values (r2=0.50; p≤0.001 for quadrant 1, r2=0.48; p≤0.001 for quadrant 2). Correlations considering the other quadrants or mesial bone loss values were weaker (r2=0.25-0.42), but significant (p≤0.008).

The risk of losing implants

A total of 295 implants were installed in 2004 in the 117 patients included in the study. There were 36.8% patients with one implant installed, 24.8% with two, 12.8% with three, 13.7% with four, 4.3% with five, and 7.7% patients with ≥6 implants installed. Of the 295 implants, 30.5% were installed in quadrant 1 (Q1), 24.7% in quadrant 2 (Q2), 19.7% in quadrant 3 (Q3) and 25.1% in quadrant 4 (Q4). Overall, positions 5 and 6 were the most frequent for implant insertion, accounting for 45.1% of all implants. Of the 295 implants, 31 (10.5%) were installed immediately after dental extraction. Regenerative techniques were used in 17.3% (51 out of 295) of implant insertions. Complete reconstruction was carried out in 27.4% patients, using fixed dental prostheses in 90.4% of the 295 implants. Reconstructions were friction-retained in 9.9% implants, cemented in 32.5% implants and screw-retained in 57.5% implants. Casted plastic burnout abutments were used in 43.2% implants, machined titanium abutments in 35.0%, and casted-on premachined palladium abutments in 21.8%.

In the 2009 revision visit, 8 out of 117 (6.8%) patients had lost implants: four patients lost one implant, three patients lost two implants and one patient lost three implants. The 13 lost implants represented 34.2% of the 38 implants installed in these eight patients in 2004. All patients that lost implants had multiple (from 3 to 6) implants installed in 2004. On a patient basis there was a tendency for significance for a greater percentage of lost implants in patients with multiple implant installed (8 out of 74 patients) versus in those with one implant installed (0 out of 43): 10.8% vs. 0% (p=0.026). This tendency disappeared when the analysis was performed on implant basis: 0 out of 43 for single implant insertion vs. 5.2% (13 out of 252) for the number of implants in patients with multiple implants installed (p=0.600).

Of the 13 out of 295 (4.4%) implants that were lost, 24.5% and 19.9% corresponded to positions 6 and 5, respectively. In Q2, 8.2% implants were lost, 6.9% in Q3, 2.7% in Q4 and 1.1% in Q1.

Is there a relationship between teeth (and their bone levels) and implant losses?

Among edentulous quadrants in 2004, the percentage of implant loss was 1.4% (1 out of 73). This percentage of implant loss increased to 3.6% (6 out of 168) in quadrants where teeth were preserved from 2004 to 2009 and to 11.1% (6 out of 54) among quadrants losing teeth in the same period (p=0.037).

The relation of implant loss with the bone loss (in the X-ray in 2004) for the associated teeth in the quadrant could be analysed in 12 of the 13 implants lost (one lost implant was in an edentulous quadrant). No significant correlation (r2=0.386; p=0.346) was found between medial bone losses for the associated teeth and implant losses, but the percentage of implant loss significantly (p<0.001) increased from 0.93% (2 out of 215) when the medial bone loss was <2 mm to 36.0% (9 out of 25) when it was ≥3 mm. Neither significant (p=0.097), although high (r2=0.626), was the correlation with distal bone loss values for associated teeth, the percentage of implant loss also significantly (p<0.001) increasing from 0.0% (0 out of 206) when the distal bone loss was <2 mm to 28.6% (6 out of 21) when it was ≥3 mm.

Periodontal health in preserved teeth

In the 335 study teeth (those identified in the panoramic X-ray performed in 2004 and remaining in 2009), mean pocket probing depths were: 3.4 ± 1.7 mm for mesio-vestibular (MV), 2.8 ± 1.6 mm for vestibular (V), 3.6 ± 1.7 mm for disto-vestibular (DV), 3.3 ± 1.6 mm for mesio-lingual (ML), 2.8 ± 1.6 mm for lingual (L), and 3.5 ± 1.6 mm for disto-lingual (DL), without differences between quadrants. A significant correlation was found between values measured at the different sites (r2≥0.71; p≤0.001). Plaque was visible in 25.3% (85 out of 335) teeth, with significant lower percentage of teeth with visible plaque in upper than in lower quadrants (19.3% vs. 32.4%; p=0.006). Mobility was described in 24.8% teeth, bleeding on probing in 59.7%, and suppuration in 2.7%. ( Table 1) shows clinical variables in preserved teeth distributed by plaque index. Those teeth presenting plaque had significantly (p<0.001) higher mesial and distal bone losses in the panoramic X-ray in 2004 and higher (p<0.001) pocket probing depths in all measuring sites in 2009. ( Table 2) shows clinical variables in 2009 in preserved teeth distributed by bone loss category in 2004. Visible plaque was significantly (p<0.001) more frequent in teeth showing ≥5 mm bone loss in 2004, that was also the group of teeth significantly (p<0.001) showing the highest pocket probing depths. Mean values of pocket probing depth in all measured sites were approximately 2-fold higher in teeth with ≥5 mm bone loss in 2004 than in those with <3 mm. Significant correlations (r2≥0.50; p<0.001) were found between values of bone loss in 2004 (both considering mesial or distal values) and values of pocket probing depths in 2009, regardless the measuring site.

**Table 1:**
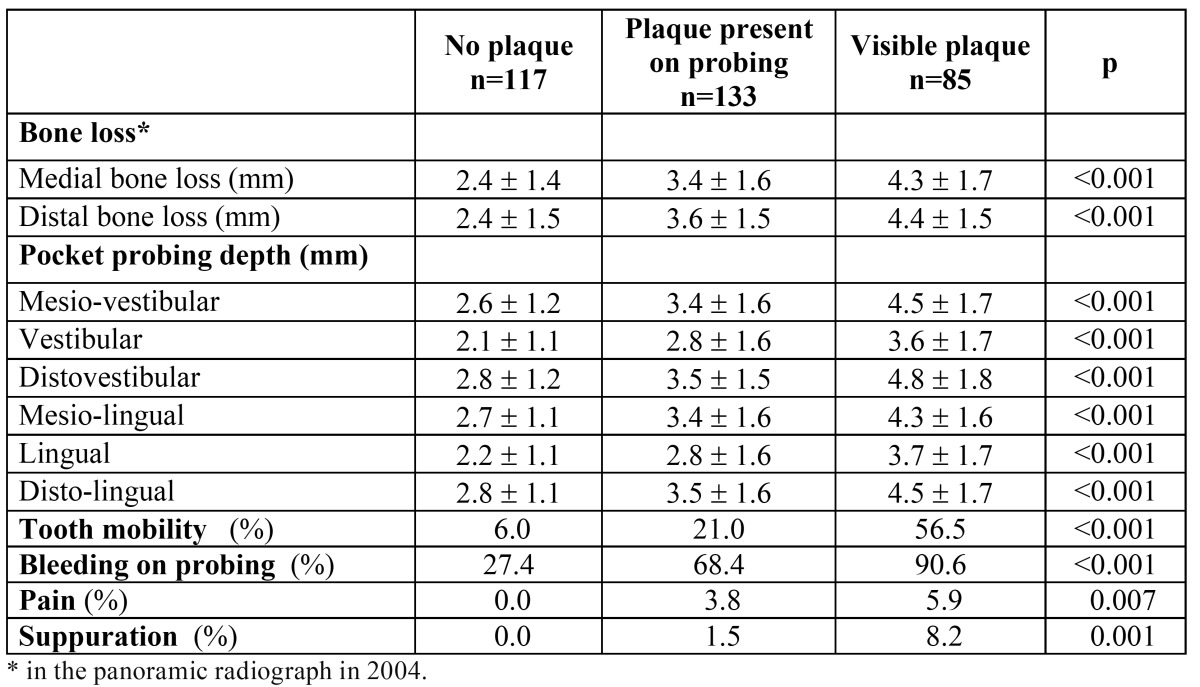
Preserved teeth. Clinical variables distributed by dental plaque index in preserved teeth.

**Table 2:**
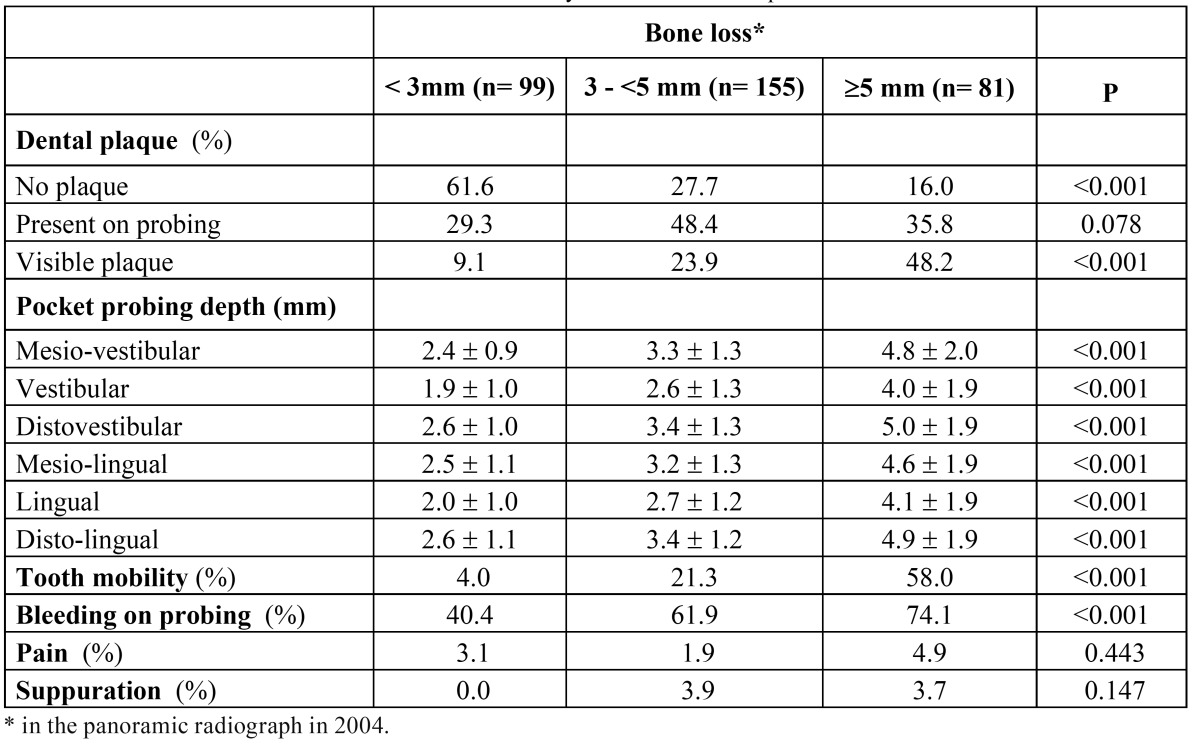
Preserved teeth. Clinical variables distributed by distal bone loss in preserved teeth.

Peri-implant health in preserved implants

Of the 282 implants that remained in function in 2009 (95.6% of the 295 installed), full data was available for 268 implants. Mean peri-implant marginal bone loss (considered as the difference between the bone level in 2004 and the bone level in the X-ray in 2009) was 0.9 ± 1.1 mm for mesial and 1.0 ± 1.2 for distal values, with significantly (p=0.001) lower values in the right side for distal measures (0.8 ± 0.9 mm for Q1+Q4 vs. 1.2 ± 1.3 mm for Q2+Q3). No significant differences were found between upper and lower quadrants.

Pocket probing depths were: 2.6 ± 1.5 mm for MV, 2.1 ± 1.2 mm for V, 2.6 ± 1.4 mm for DV, 2.6 ± 1.4 mm for ML, 2.2 ± 1.3 mm for L, and 2.6 ± 1.4 mm for DL. A significant correlation was found between values measured at the different sites (r2≥0.68; p≤0.001). Values in the right side tended to be higher than those in the left side for MV (2.7 ± 1.3 mm vs. 2.3 ± 1.4; p=0.028).

Plaque was visible in 13.4% (36 out of 268) implants, with lower percentage of implants with visible plaque in right than in left quadrants (9.5% vs. 17.8%; p=0.04). Bleeding on probing was found in 54.9% implants. ( Table 3) shows clinical variables in preserved implants distributed by plaque index. Those implants presenting plaque had significantly (p<0.001) higher mesial and distal bone losses, higher (p<0.001) pocket probing depths in all measuring sites, and higher percentage of bleeding on probing.

**Table 3:**
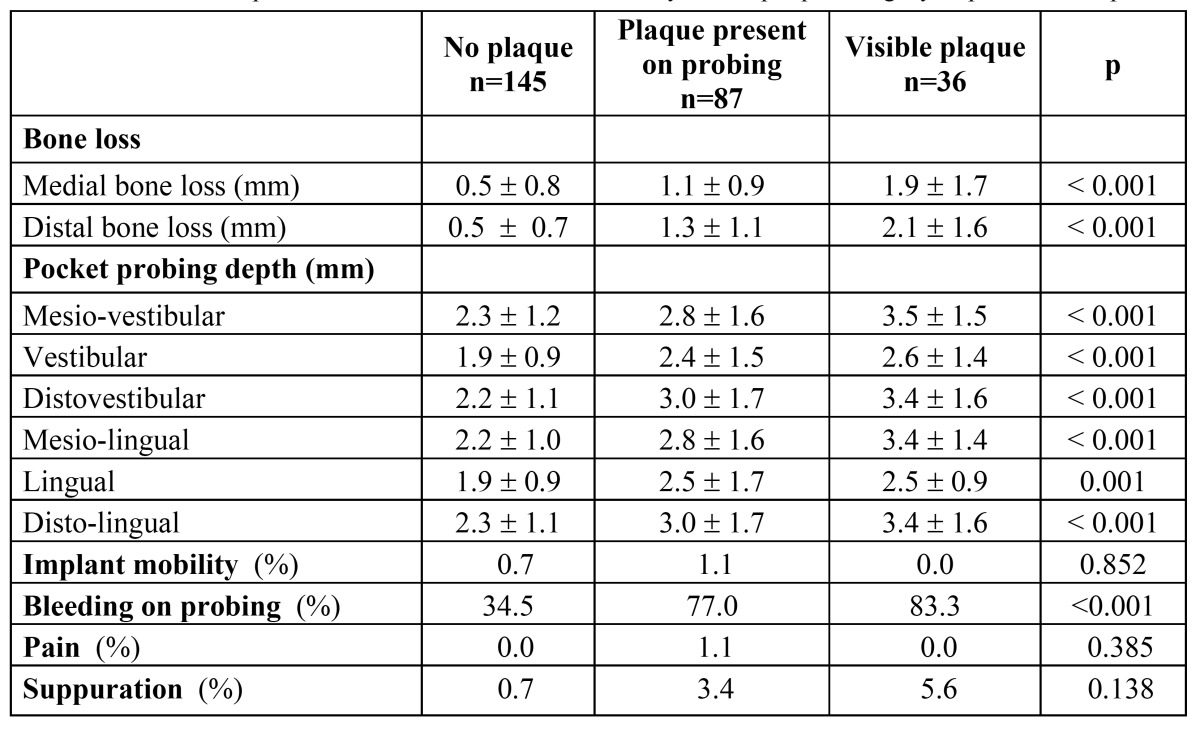
Preserved implants. Clinical variables distributed by dental plaque category in preserved implants.

 Table 4 shows clinical variables in preserved implants distributed by bone loss. Only 15 (5.6%) implants showed ≥3 mm of distal bone loss. Implants showing 2 or ≥3 mm bone loss showed more frequently (p<0.001) plaque, bleeding on probing, and the highest pocket probing depths (p<0.001). Weak but significant correlations (r2=0.34; p<0.001) were found between values of bone loss (both considering mesial or distal values) and pocket probing depths (only when considering DV values). No differences were found in mean pocket depths between those implants with distal bone loss ≤1 mm and those with >1-<2 mm, regardless the measuring site. Differences in pocket probing depths were found between implants with ≤1 mm distal bone loss and those with 2 mm (with respect to DV, ML, L and DL measuring sites) and with ≥3 mm (all measuring sites). Pocket probing depths of implants with 2 mm bone loss were also statistically different from those of implants with ≥3 mm bone loss (in all measuring sites except L).

**Table 4:**
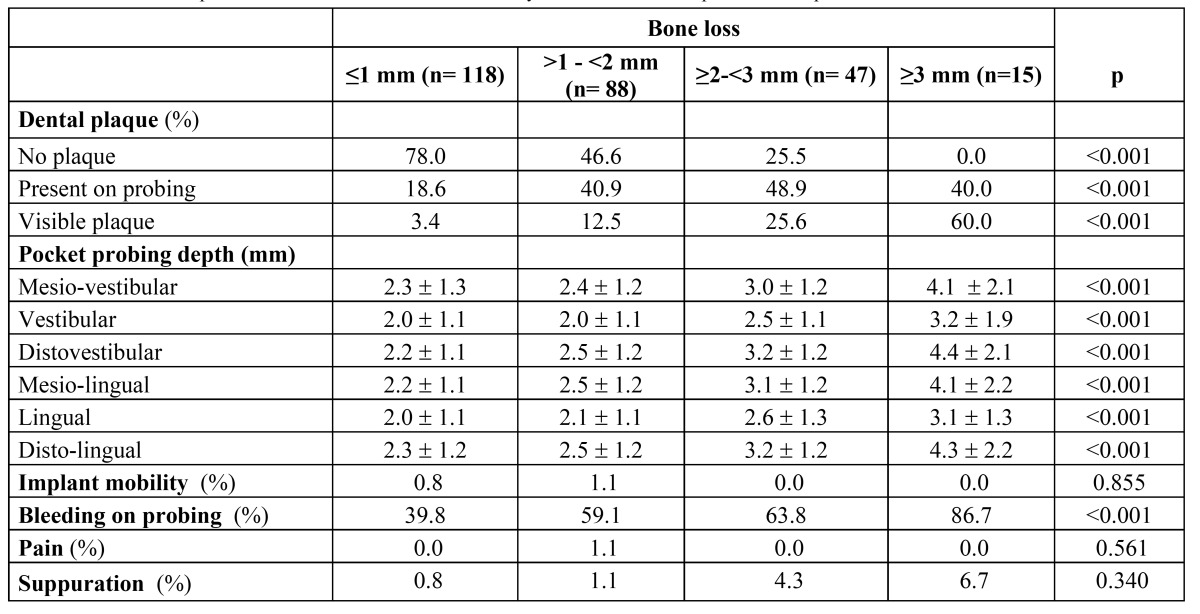
Preserved implants. Clinical variables distributed by distal bone loss in preserved implants.

Is there a relationship between clinical parameters from preserved teeth and implants? 

Significantly higher percentage of teeth than implants presented plaque (grouping plaque visible + plaque present by probing): 65.1% vs. 45.9%; p<0.001 ( Table 1 and  Table 3) and plaque visible (25.4% vs. 13.4%; p<0.001). Significant (p<0.001) higher values of bone loss (both mesial and distal) and pocket probing depths (in all measuring sites) were found in teeth vs. implants. While pocket probing depths (regardless the measuring site) were similar in teeth and implants with no plaque ( Table 1 and  Table 3), pocket probing depths were significantly higher for teeth with visible plaque than for implants with visible plaque (p<0.01). Similarly, while no differences were found between pocket probing depths (regardless the measuring site) in teeth and implants in the lowest bone loss categories ( Table 2 and  Table 4), pocket probing depths were significantly higher (p<0.001) for teeth with ≥5 mm bone loss than for implants with bone losses of ≥2 - <3 mm, but were similar for implants with bone losses ≥3 mm (p>0.06).

Significant (p<0.001) correlations in pocket probing depths were found between implants and the associated teeth in the same quadrant: r2=0.35 for MV, r2=0.55 for V, r2=0.36 for DV, r2=0.40 for ML, r2=0.47 for L, and r2=0.36 for DL.

The role of bone loss and plaque index on pocket probing depths

For teeth, the multivariate analysis was significant (p<0.001), with r2 ranging from 0.35 to 0.47 depending on the pocket depth measuring site. Pocket probing depths were significantly correlated with plaque index (β from 0.118 to 0.227 -depending on the measuring site-; p<0.001) and bone loss (in mm; β from 0.512 to 0.598 -depending on the measuring site-; p<0.001).

In the case of implants, although statistical significance was reached (p<0.001) in the model, the overall predictability was lower (r2 from 0.055 to 0.218). Pocket probing depths were significantly correlated with bone loss (in mm; β from 0.207 to 0.405 -depending on the measuring site-; p≤0.002) but not with plaque index (β from 0.056 to 0.144 -depending on the measuring site-; p≥0.02).

## Discussion

In Odontology disease definitions rely on measures of probing depth, bone loss and bleeding on probing (15). In the current study the analysis of periodontal health (based on these measures) showed a significant per-quadrant and inter-quadrant correlation between values of bone loss as well as between all pocket depth values, indicating a generalized healthy/unhealthy situation in the studied patients. The significant and bi-directional relation between plaque and bone loss, and between plaque/bone loss with pocket probing depths, demonstrates the interrelationship of the parameters/signs used for dental assessment in daily practice. However the critical issue is which parameter/sign and value should be used as best predictor for disease. In our study bone losses of ≥5 mm in the panoramic X-ray performed in 2004 were significantly associated with presence of visible plaque, higher pocket depths in all sites, higher bleeding on probing and higher mobility when teeth were assessed in 2009, that is, a significant association with higher values for the parameters used to evaluate the periodontal lesion. Even more, the bone loss recorded in 2004 could be related with the risk of losing teeth, with >50% risk associated with distal bone losses ≥7 mm, and an adjusted odds ratio of 1.60 per mm. These results suggest not only that bone loss could be used as parameter predictive for losing teeth, but also they suggest a cutoff value of ≥7 mm for a probability >50% of losing teeth.

Some authors indicate that patients with periodontitis may experience more implant loss than non-periodontitis patients (5,16,17) based on the etiological similarities in the pathogenesis of periodontitis and peri-implantitis, although there is limited evidence for it (5). Bacterial colonization at peri-implant sulcus in newly inserted implants occurs rapidly (18), and periodontal pockets of teeth may act as reservoir for microorganisms to colonize the newly inserted implants (18,19). Although the number of lost implants in this study is small and thus data should be taken with caution, our results showed a tendency toward lower rates of implant loss in edentulous quadrants than in those where teeth were preserved, with quadrants losing teeth between 2004 and 2009 showing the highest implant loss rate. Even more, regardless the correlations between bone losses for associated teeth and implant losses, although high were not significant, the percentage of implant loss was significantly higher when the bone loss for the associated teeth was ≥3 mm. With respect to the peri-implant lesion, significant (p<0.001) correlations were found in pocket probing depths between implants and the associated teeth in the same quadrant. Although globally significantly higher values of marginal bone loss and pocket probing depths were found in teeth vs. implants, similar pocket depths were found in implants with ≥3 mm of distal bone loss and in teeth with ≥5 mm of distal bone loss, with high values in both cases, and with significant correlations between all measuring sites between implants and teeth. This reinforces, from the clinical point of view, the similar pathogenesis of perio-dontitis and peri-implantitis, and suggests that the associated marginal teeth bone loss in 2004 could potentially be considered as risk marker for the peri-implant lesion in 2009. This similar pocket depths in teeth and implants with lower marginal bone loss for implants (≥3 mm vs. ≥5 mm) ( Table 2 and  Table 4) is in accordance with reports indicating that peri-implant soft tissues seem to be more rapidly destroyed than dental tissues (20) that had been exposed to microbial challenge for decades (21). This suggests that the periodontal status of the patient is a critical issue in predicting the survival of the new inserted implant.

Another interesting finding was that, on a patient basis, there was a tendency for significance for a greater percentage of lost implants in patients with multiple implant installed versus in those with one implant installed (10.8% vs. 0%). This could be interpreted as consequence of the worse periodontal status of patients receiving more than one implant, confirming once more the analogy of periodontitis and peri-implantitis and the global healthy/unhealthy per-patient condition in the studied patients. However there is a strong confounding factor (more implant loss when more implants are inserted) since when the analysis is performed on implant basis, the tendency disappeared since there were not differences between implant lost in single installations (0%) and in multiple installations (4.4%).

Several definitions of peri-implant disease are found in the literature (5,16,21,22). Considering bleeding on probing as sign for peri-implant mucositis in the absence of bone loss, we can approximate to 39.8% the prevalence of peri-implant mucositis (on implant basis) in the present study since it was the percentage of implants with bone loss ≤1 mm presenting bleeding on probing ( Table 4). This prevalence is lower than the >50% prevalence (on implant basis) reported by other authors (21,23,24). Definitions of peri-implantitis in the literature vary depending on the amount of bone loss used in the definition, with most of them considering ≥2.5- ≥3 mm as cutoff for definition (5,21,25). Albrektsson et al. considered a non pathological bone loss of around 0.5 mm the first year of service, and that annually the vertical bone loss should not exceed 0.2 mm (26). According to this criterion, non pathological bone loss 4-5 year after implant insertion would be 1.1-1.3 mm. Therefore in the present study implants showing >1.1 mm of bone loss and bleeding on probing should be defined as unhealthy implants. As shown in  Table 4, 150 out of 268 (56.0%) implants presented bone losses >1 mm, associated in 95 of them with bleeding on probing. Therefore we can approximate the prevalence of peri-implantitis to 35.4% (95 out of 268) that is within the range (12-40% sites) of data supplied by the pooled analysis performed with studies on implants in function for ≥5 years (21). Nevertheless when considering all daily practice parameters/signs (plaque, six measures of pocket depths and bleeding on probing) ( Table 4) it should be noted that values for implants with >1-<2 mm of bone loss were closer to values for implants with ≤1 mm of bone loss, and significantly different from those for implants with ≥3 mm of bone loss that showed much higher values (mean pocket depths of approx. 4 mm, 60% visible plaque and 86.7% bleeding on probing). According to this, the first 2 millimeters of bone loss could be attributed to the establishment of the biological width, not necessarily related to peri-implant disease when other clinical parameters are in concordance with health condition, and the clinical cutoff would be ≥3 mm of bone loss. With this criterion, the prevalence of peri-implantitis (implants with ≥3 mm of bone loss showing bleeding on probing) in our study would be 4.9% (13 out of 268 implants) 4-5 years post-implant installation.

The relative importance of microbial factors and mechanical forces as cause of peri-implantitis remains controversial. Through performing an explicative multivariate analysis we tried to determine the role of bone loss and plaque index (as independent variables) on pocket probing depths (as dependent variable), both for teeth and implants. Significant correlations were found for both independent variables and pocket depths in teeth, but in implants significant correlations were only found for bone loss. The discussion on the importance of microbial and mechanical factors in the depth of pockets as well as for bone loss may be academic since in clinical practice, as shown in this study, bone loss is associated with deepening of pockets that intuitively create an increasing anaerobic environment colonised by intra-oral translocation of periodontal pathogens from teeth showing chronic periodontitis. This may contribute to a further marginal bone loss (19), although this remains to be proven.

Practice-based dental research network studies based on clinical parameters/signs used in daily practice may be helpful in investigating indices for prediction of teeth survival (thus indicating when to insert an implant) and the survival of implants. The results of the present study, where the periodontal status was assessed by clinical parameters of the tooth with the highest bone loss at baseline, showed the significant bi-directional relation between plaque and bone loss, and between each of these two parameters/signs with pocket depths and bleeding on probing, both in teeth and implants, and between them. This fact and the higher percentage of implants lost when the bone loss of the associated teeth was ≥3 mm reinforces the idea that the periodontal status of the patient is a critical issue in predicting implant health/lesion and that parameters measured in daily practice (bone loss, plaque index, pocket depth and bleeding on probing) may provide quantitative values for assessing longevity of implants. Further prospective studies are needed to conclusively confirm correlations, especially in relation to cutoff values of bone loss to define peri-implantitis.
